# Resurgence to Life: A Case Report on Inpatient Rehabilitation in Organophosphate Poisoning Followed by Intermediate Syndrome

**DOI:** 10.7759/cureus.49069

**Published:** 2023-11-19

**Authors:** Pallavi R Bhakaney, Vaishnavi D Yadav, Aditi A Nagore, Chaitanya A Kulkarni

**Affiliations:** 1 Cardiorespiratory Physiotherapy, Dr. D. Y. Patil College of Physiotherapy, Pune, IND; 2 Cardiorespiratory Physiotherapy, Ravi Nair Physiotherapy College, Datta Meghe Institute of Higher Education and Research, Wardha, IND; 3 Community Physiotherapy, Dr. D. Y. Patil College of Physiotherapy, Pune, IND

**Keywords:** case report, rehabilitation, physiotherapy, intermediate syndrome, organophosphate poisoning

## Abstract

Organophosphate poisoning (OPP) results from the occupational, accidental, or suicidal intake of organophosphate pesticides (OPs). There is a huge limitation in the available literature on OPP cases and the role of physiotherapy in such cases. We report a case of a 33-year-old farmer, found in a state of ingested insecticide and admitted to the emergency department. The patient had two to three episodes of vomiting, associated with continuous tremors in his hands and legs. Immediately infused with atropine, the patient's general condition deteriorated, and he was intubated with an endotracheal tube. With the signs of long-term intubation, a tracheostomy was done. A respiratory therapy consult was taken for indications of intermediate syndrome and to achieve early weaning. The patient's referral was received in view of the long-term requirement of bronchial hygiene and the need for early ambulation as well as psychological support. Informed consent was taken from the family prior to the commencement of the treatment. Respiratory therapy interventions included body positioning, bronchial hygiene techniques, chest proprioceptive neuromuscular facilitation (PNF) techniques, and mobility exercises to achieve early ambulation. Physiotherapists have the appropriate training, knowledge, and skills to deliver the exercise components and help patients return to their activities of daily living. Significant levels of improvement have been seen in the general condition of the patient. The overall functioning of the patient's health was seen as improved on the scales of consciousness, early mobility in the step-down unit, and quality of life.

## Introduction

Organophosphate poisoning (OPP) results from the occupational, accidental, or suicidal intake of organophosphate pesticides (OPs) [1]. In developing countries, OPs are widely available and used to commit suicide in farming areas, less commonly by accident [2]. The mortality rate of OPP is higher in developing countries. The usage of OPs or self-poisoning agents is a clinical problem in rural regions of the developing world and kills an estimated 250,000 people every year [3]. Because of its low cost and easy availability, organophosphate becomes the agent of choice for self-poisoning [4]. OPs are commonly used for farming in India, which is mostly agrarian. Globally, pesticide poisoning from OPs is a serious occupational hazard worldwide, accounting for more than 80% of pesticide-related deaths and hospitalizations [5]. Because India is an agriculturally based country, OPs remain the primary crop protection and pest control chemical [6]. Organophosphate is used in insecticides, medications, and nerve agents. Poisoning is a major public health issue that affects people all around the world [7]. Signs and symptoms of OPP are classified into acute (minutes to 24 hours), delayed (24 hours to two weeks), and late (beyond two weeks) onset [8]. Symptoms include increased salivation and tear production, diarrhea, vomiting, small pupils, sweating, muscle tremors, and confusion [9]. Poisoning with OPs is linked to several conditions, including intermediate syndrome (IMS) and organophosphate-induced postponed neuropathy (OPIPN) [10]. Following the oral ingestion of OP insecticides, IMS occurs in about 20% of individuals [11]. It is a delayed onset of muscle weakness and paralysis [12] and develops after 24-96 hours as a result of acetylcholine's prolonged action at nicotinic receptors, resulting in ocular, neck, limb, and respiratory muscular weakness [13]. Delayed neuropathy is a sensory-motor peripheral neuropathy characterized by distal motor weakness with the involvement of cranial nerves, and proximal muscles, as well as distal sensory symptoms including paresthesia or numbness [14]. OPP can result in several significant side effects [15]. These include metabolic disorders including hyperglycemia (high blood sugar) and glucosuria (excess sugar in the urine), diabetic ketoacidosis (excess blood acids), respiratory failure, seizures, aspiration pneumonia, delayed neuropathy, and death [16]. There is a limited literature that shows the effectiveness of physiotherapy rehabilitation in such cases of OPP, and this case has been documented to exhibit the significant yet important role of physiotherapy in these cases. This case report aims to highlight the successful role of physiotherapy in managing OPP, focusing on the rehabilitation of patients with OPP-related muscular weakness and paralysis.

## Case presentation

A 33-year-old farmer was admitted to medicine intensive care unit (ICU) after being brought to casualty in a disoriented state. His relative reported that he had been found unconscious near their farm, with excessive mouth secretions and profuse sweating. He also noticed a bottle of an insecticide at his side, which might have been taken under the influence of alcohol as the patient is an occasional alcoholic. Immediately, they brought the patient to our tertiary care hospital. On their way to the hospital, the patient had two to three episodes of vomiting which was greenish, along with continuous tremors in his hands and legs. The reported history by the relative revealed that the patient was a chronic alcoholic for 15 years. On admission, necessary investigations were done, and medical management was started, which included injection of atropine 2 ml/hour, injection of PAM (Acme Pharmaceuticals, Ahmedabad, Gujarat, India) 20 ml/hour, injection of C-Tri (Zuventus Healthcare Ltd, Pune, Maharashtra, India), injection of PAN (Alkem Laboratories Ltd, Mumbai, Maharashtra, India), injection of Emeset (Cipla Ltd, Mumbai, Maharashtra, India), and intravenous fluids. As per his symptoms, the dose of atropine was usually given as the initial loading dose of 2 mg followed by infusion at the rate of 5 mg/hour and repeated every five to ten minutes. After two hours, the patient started presenting with mental obfuscation. He was intubated immediately with an endotracheal tube with volume control mode as he was presenting with tachypnea (respiratory rate: 40 breaths/min) and arterial blood gases (ABG) showed arterial oxygen tension of less than 50 mmHg on room air. After eight days, the patient underwent a tracheostomy given prolonged ventilation. A physiotherapy referral was given because of former issues along with the prevention of further complications. The course of ventilator settings is shown in Table 1.

**Table 1:** Course of ventilator settings VCM: volume control mode; SIMV: synchronized intermittent mandatory ventilation mode; FiO_2_: fraction of inspired oxygen; PEEP: positive end-expiratory pressure; pH: potential of hydrogen; PaO_2_: partial pressure of arterial oxygen; PaCO_2_: partial pressure of arterial carbon dioxide; HCO_3_: bicarbonate; O_2_: oxygenation; mmHg: millimeters of mercury; mmol/L: millimole per liter

Week	Ventilator mode	Ventilator setting	Arterial blood gases
Week 1	VCM via endotracheal tube	FiO_2_, 80%; PEEP, 5	pH, 7.481 mmHg; PaO_2_, 215 mmHg; PaCO_2_,46.9 mmHg; HCO_3_, 26.1 mmol/L
Week 2	SIMV via tracheostomy	FiO_2_, 80%; PEEP, 5	pH, 7.471 mmHg; PaO_2_, 200 mmHg; PaCO_2_, 45.8 mmHg; HCO_3_, 25.5 mmol/L
Week 2	VCM	FiO_2_, 70%; PEEP, 5	pH, 7.461 mmHg; PaO_2_, 197 mmHg; PaCO_2_, 44.7 mmHg; HCO_3_, 24.1 mmol/L
Week 3	SIMV + pressure support	FiO_2_, 70%; PEEP, 5	pH, 7.459 mmHg; PaO_2_, 196 mmHg; PaCO_2_, 43.6 mmHg; HCO_3_, 23.1 mmol/L
Week 3	Continuous positive airway pressure + pressure support	FiO_2_, 50%; PEEP, 5	pH, 7.457 mmHg; PaO_2_, 194 mmHg; PaCO_2_, 41.1 mmHg; HCO_3_, 22.3 mmol/L
Week 4	Spontaneous breathing trial	8 liters of O_2_	pH, 7.373 mmHg; PaO_2_, 80 mmHg; PaCO_2_, 38 mmHg; HCO_3_, 25.2 mmol/L

**Figure 1:**
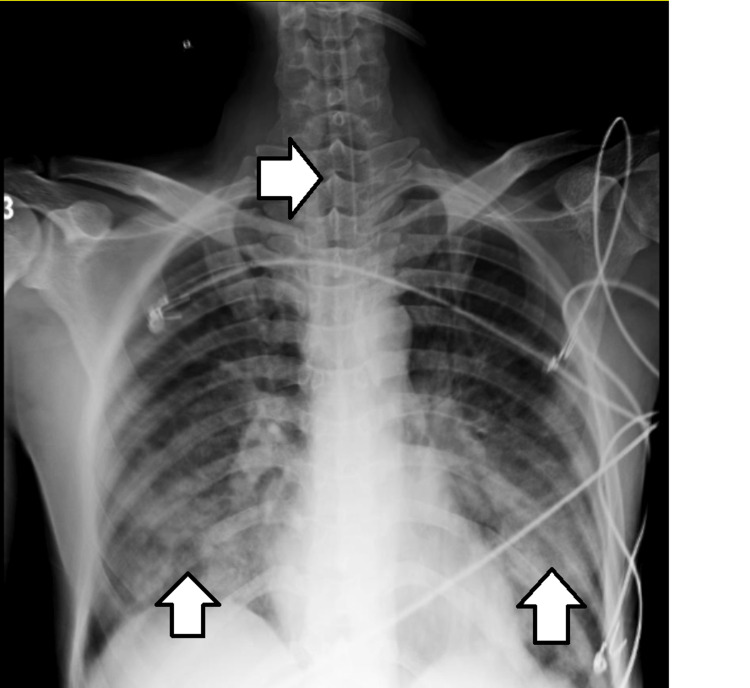
Impression of an endotracheal tube along with bilateral heterogeneous opacities at lower zones

During examination, the patient was in a supine lying position, unconscious with poor general condition and excessive oral secretions. On neurological examination, his Glasgow Coma Scale (GCS) score was 6/15. On observation, an endotracheal tube was present in situ connected to a mechanical ventilator, with mode-volume control, fraction of inspired oxygen (FiO_2_) of 80%, positive end-expiratory pressure (PEEP) of 5 cmH_2_O, and cuff pressure of 25 mmHg. Vital signs were a pulse rate of 120 beats/minute, blood pressure of 150/90 mmHg, and respiratory rate of 33 breaths/minute. Ryle's tube and Foley's catheter were also seen. Respiratory examination revealed decreased chest movements bilaterally and the presence of bilateral crepitations at axillary and mammary levels. Deep tendon reflexes were found to be sluggish, i.e., 1+ grading. Pinpoint pupils were present. His muscle power on the Medical Research Council (MRC) Scale for Muscle Strength indicated grade 3 in the upper limb and grade 2 in the lower limb, and no sensory symptoms were present. Laboratory investigations at the time of admission showed normal renal functions, liver functions, and normal serum levels of sodium, potassium, calcium, and magnesium, but serum cholinesterase was 6.7. The radiological investigation included chest X-rays (antero-posterior view), which showed the impression of an endotracheal tube along with bilateral heterogeneous opacities at lower zones, along with impressions of leads recording the electrical function of the heart (Figure 1).

**Figure 2:**
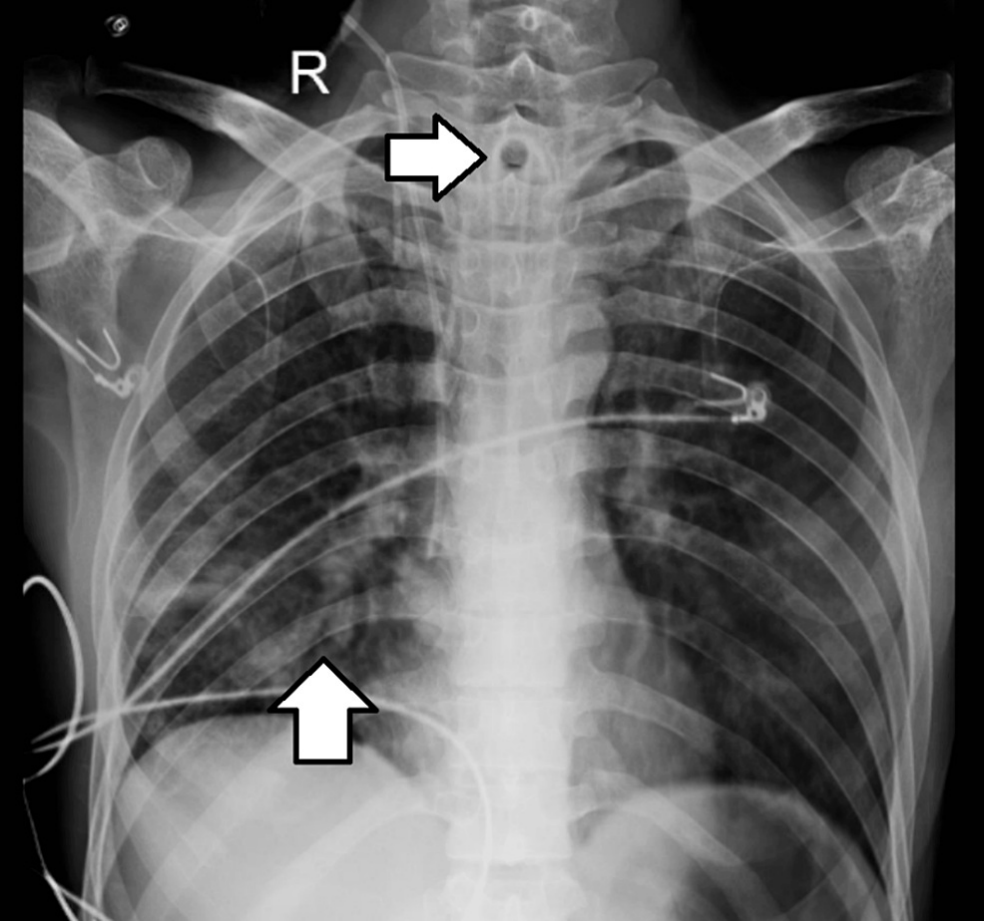
Impression of a tracheostomy tube in situ with heterogeneous opacities seen in the right lower zones

Another chest X-ray (antero-posterior view) showed an impression of a tracheostomy tube in situ with heterogeneous opacities seen in the right lower zones, along with impressions of leads recording the electrical function of the heart (Figure 2).

The patient was diagnosed with OPP, with a severity categorized as minor on the Poisoning Severity Score, associated with IMS. The primary goal of the physiotherapist was to begin the process of weaning through bronchial hygiene and a spontaneous breathing trial, which was achieved by body positioning, percussion, and vibration techniques for ten minutes, four hourly followed by suctioning. Suctioning was done using a 12Fr-sized catheter, and the suction pressure was maintained at 120 mmHg. Infection control protocol was followed to prevent further complications related to hospital-acquired diseases. The patient was positioned in a side-lying position every two hours. To prevent the further deterioration of lung function, a ventilator-associated pneumonia (VAP) bundle was followed. The VAP bundle care included maintenance of intra-cuff pressure values, hand hygiene, daily oral hygiene, personal protective equipment for suctioning, placement of patients in semi-recumbent position with the head elevated at 30^o^, daily cleaning of the ventilator, suction bottle with sterile distilled water, and sterilization of circuit by the nursing staff. Manual hyperinflation, manual chest percussion and compression, rib springing, postural drainage, chest vibrations, and suctioning administration are proven to be effective physiotherapy techniques in changing respiratory mechanics in VAP patients. The patient was taken on a tracheostomy tube and given prolonged ventilator requirements due to pneumonitis and IMS on the second week of admission. The primary goal of physiotherapy was to facilitate the patient's weaning from mechanical ventilation and address bronchial hygiene, while the secondary goal involved maintaining muscle integrity and mobility. Disconnected from the mechanical ventilator on week 4, the patient was able to maintain spontaneous breathing for 48 hours followed by no further requirement of the ventilator. The same intervention was continued twice daily with four hourly suctioning. The secondary goal was to maintain muscle integrity along with mobility. Passive limb mobility exercises to the shoulder, elbow, wrist, hip, knee, and ankle joints were given to maintain joint integrity. Along with mobility exercises, joint compressions were also given to improve the proprioceptive input. The mobility exercises were explained and demonstrated to the relative and were advised to be done immediately between the physiotherapy sessions. The patient progressed from bedside sitting to standing to spot walking. The frequency of exercises was fully controlled by the patient to avoid the deteriorating effect of training. Spot marching was followed by walking with maximal support which progressed to minimal support as strengthening was started. Strengthening was started for the major muscle groups with 0.5 kg dumbbells in week 5. It was performed once a day, under the careful monitoring and supervision of the therapist. Throughout week 6 and week 7, the protocol was continued incorporated with alternate-day aerobic training. It included walking on high knees with minimal assistance, sit-to-stand, step-up and step-down, and walking around the hallway as tolerated by the patient. The effectiveness of the intervention was measured using relevant outcome measures mentioned in Table 2.

**Table 2:** Outcome measures ICU: intensive care unit

Scales	Week 1	Week 4	Week 7
Glasgow Coma Scale	2/15	10/15	15/15
ICU Mobility Scale	0 (lying on the bed)	4 (standing)	8 (walking with the assistance of one person)
Functional Independence Measure Scale	Maximal assistance	Moderate assistance	Minimal assistance
Hospital Anxiety Depression Scale	Severe depression and anxiety (Score-15)	Moderate depression and anxiety (Score-9)	Moderate depression and anxiety (Score-8)

## Discussion

This case study showed the effectiveness of physiotherapy rehabilitation in patients with OPP. Poisoning with OP is linked to several conditions, including IMS and OPIPN [17]. Following oral ingestion of OP insecticides, IMS occurs in about 20% of individuals [18]. It is a delayed onset of muscle weakness and paralysis and develops after 24-96 hours due to acetylcholine's prolonged action at nicotinic receptors, resulting in ocular, neck, limb, and respiratory muscular weakness [19]. In physiotherapy management, body positioning, percussion, vibration techniques, suctioning, manual hyperinflation, rib springing, postural drainage, chest vibrations, chest proprioceptive neuromuscular facilitation (PNF) techniques, i.e., intercostal stretch and co-contraction of the diaphragm, passive limb mobility exercises to the shoulder, elbow, wrist, hip, knee, and ankle joints, and joint compressions to all peripheral joints were given to improve lung capacity, passive range of motion, and limb mobility, decrease the risk of tightness or fatigue of the respective part of the body, remove secretions, and reduce the risk of bedsore, respectively. Sneha et al. conducted a study on the effect of respiratory PNF technique with chest physiotherapy in mechanically ventilated organophosphorus poisoning patients in which physiotherapy along with PNF technique in the management of mechanically ventilated patients with pulmonary complications proved efficient for preventing pulmonary complications, clearing the mucous secretions, and better prognosis in patients with OPP. The above study emphasized on various physiotherapy regimes such as intercostal stretch which was used to improve oxygen saturation. Moreover, it also documented the lung function in terms of static and dynamic compliance which they found to have a significant difference, which implies that physiotherapy treatment is effective in improving lung function. As a measure of pulmonary function, the authors also documented the effect of physiotherapy regime on minute ventilation which they found to have a significant difference [20]. Limited literature is available on the importance of physiotherapy in such cases. Thus, there is a need to document such more evidences, which might also include the importance of psychological support. 

## Conclusions

An integrative approach of healthcare professionals plays a pivotal role in improving the health status as well as the psychological state of a patient with OPP with IMS. Physiotherapy interventions have shown remarkable efficacy in improving a patient's respiratory function as well as quality of life in this condition. There is limited literature available regarding the efficiency of physiotherapy rehabilitation in such cases. This case study helped healthcare professionals gain a better understanding in view of physiotherapy while approaching a patient with OPP.
